# Creation of Boron Vacancies in Hexagonal Boron Nitride Exfoliated from Bulk Crystals for Quantum Sensing

**DOI:** 10.1021/acsanm.3c03395

**Published:** 2023-11-28

**Authors:** Ty Zabelotsky, Sourabh Singh, Galya Haim, Rotem Malkinson, Shima Kadkhodazadeh, Ilya P. Radko, Igor Aharonovich, Hadar Steinberg, Kirstine Berg-Sørensen, Alexander Huck, Takashi Taniguchi, Kenji Watanabe, Nir Bar-Gill

**Affiliations:** † The Center for Nanoscience and Nanotechnology, The Hebrew University of Jerusalem, Jerusalem 91904, Israel; ‡ The Institute of Applied Physics, The Hebrew University of Jerusalem, Jerusalem 91904, Israel; § The Racah Institute of Physics, The Hebrew University of Jerusalem, Jerusalem 91904, Israel; ∥ School of Physics, The University of Melbourne, Parkville, Victoria 3010, Australia; ⊥ DTU Nanolab, Technical University of Denmark, Fysikvej, Kongens, Lyngby, 2800, Denmark; # Department of Physics, Technical University of Denmark, Kongens, Lyngby, 2800, Denmark; ¶ School of Mathematical and Physical Sciences, University of Technology Sydney, Ultimo, New South Wales 2007, Australia; ∇ ARC Centre of Excellence for Transformative Meta-Optical Systems (TMOS), Faculty of Science, University of Technology Sydney, Ultimo, New South Wales 2007, Australia; ○ Department of Health Technology, Technical University of Denmark, Kongens, Lyngby 2800, Denmark; ⧫ Center for Macroscopic Quantum States (bigQ), Department of Physics, Technical University of Denmark, 2800 Kongens, Lyngby, Denmark; †† International Center for Materials Nanoarchitectonics, National Institute for Materials Science, 1-1 Namiki, Tsukuba 305-0044, Japan; ‡‡ Research Center for Functional Materials, National Institute for Materials Science, 1-1 Namiki, Tsukuba 305-0044, Japan

**Keywords:** hBN, boron vacancy defects, ion implantation, electron irradiation, spin resonance, optical spectroscopy

## Abstract

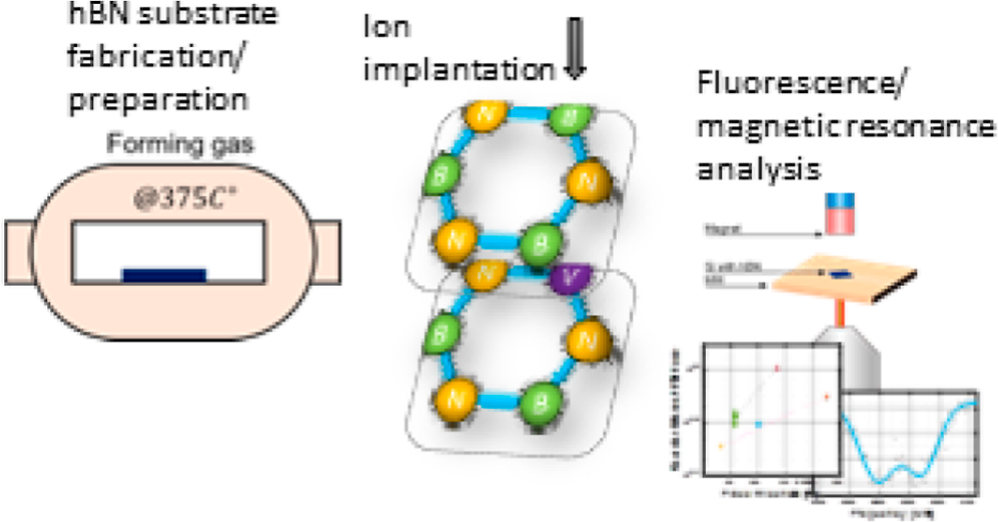

Boron vacancies (VB^–^) in hexagonal boron -nitride (hBN) have sparked great interest in recent years due to their optical and spin properties. Since hBN can be readily integrated into devices where it interfaces a huge variety of other 2D materials, boron vacancies may serve as a precise sensor which can be deployed at very close proximity to many important materials systems. Boron vacancy defects may be produced by a number of existing methods, the use of which may depend on the final application. Any method should reproducibly generate defects with controlled density and desired pattern. To date, however, detailed studies of such methods are missing. In this paper, we study various techniques for the preparation of hBN flakes from bulk crystals and relevant postprocessing treatments, namely, focused ion beam (FIB) implantation, for creation of VB^–^s as a function of flake thickness and defect concentrations. We find that flake thickness plays an important role when optimizing implantation parameters, while careful sample cleaning proved important to achieve consistent results.

## 1 Introduction

Hexagonal boron-nitride (hBN) is a graphite-analogue layered material with a hexagonal lattice where boron and nitrogen atoms alternate in each hexagon. hBN is a wide band-gap insulator, which has become an essential ingredient in stacked van der Waals (vdW) heterostructures.^1^ hBN was first used in vdW heterostructures as an ultraflat, electrically stable substrate,^2^ and as a means to fully encapsulate graphene.^3^ It was soon realized that ultrathin hBN flakes can be used as tunnel barriers^4^ and that hBN capping can stabilize air-sensitive materials.^5^ Since hBN has strong adhesion to graphene, it has enabled the use of selective tearing and stacking, the key method used in the assembly of twist-angle-controlled devices.^6^

Defects in hBN have been studied using electronic transport - where they are identified as nanoscale quantum dots, sensitive to the spectra and compressibility of nearby samples.^7,8^ They have also been extensively studied by using optical fluorescence. Color centers in hBN, specifically VB^–^s, are stable spin defects in the hBN lattice with similar traits to the well-known nitrogen vacancies (NV^–^) in diamond^9−12^ and have comparable applications.^13^

Given the potential of such optically active spin defects in vdW materials, significant effort has been invested over the past few years in the controlled fabrication and characterization of these color centers.^14−16^ Nevertheless, despite the significant literature on the subject, robust and controlled defect creation still poses a challenge, and a systematic study of VB^–^ creation as a function of method (e.g., electron irradiation, ion implantation), preparation protocol, and flake thickness is still lacking.

**Figure 1:**
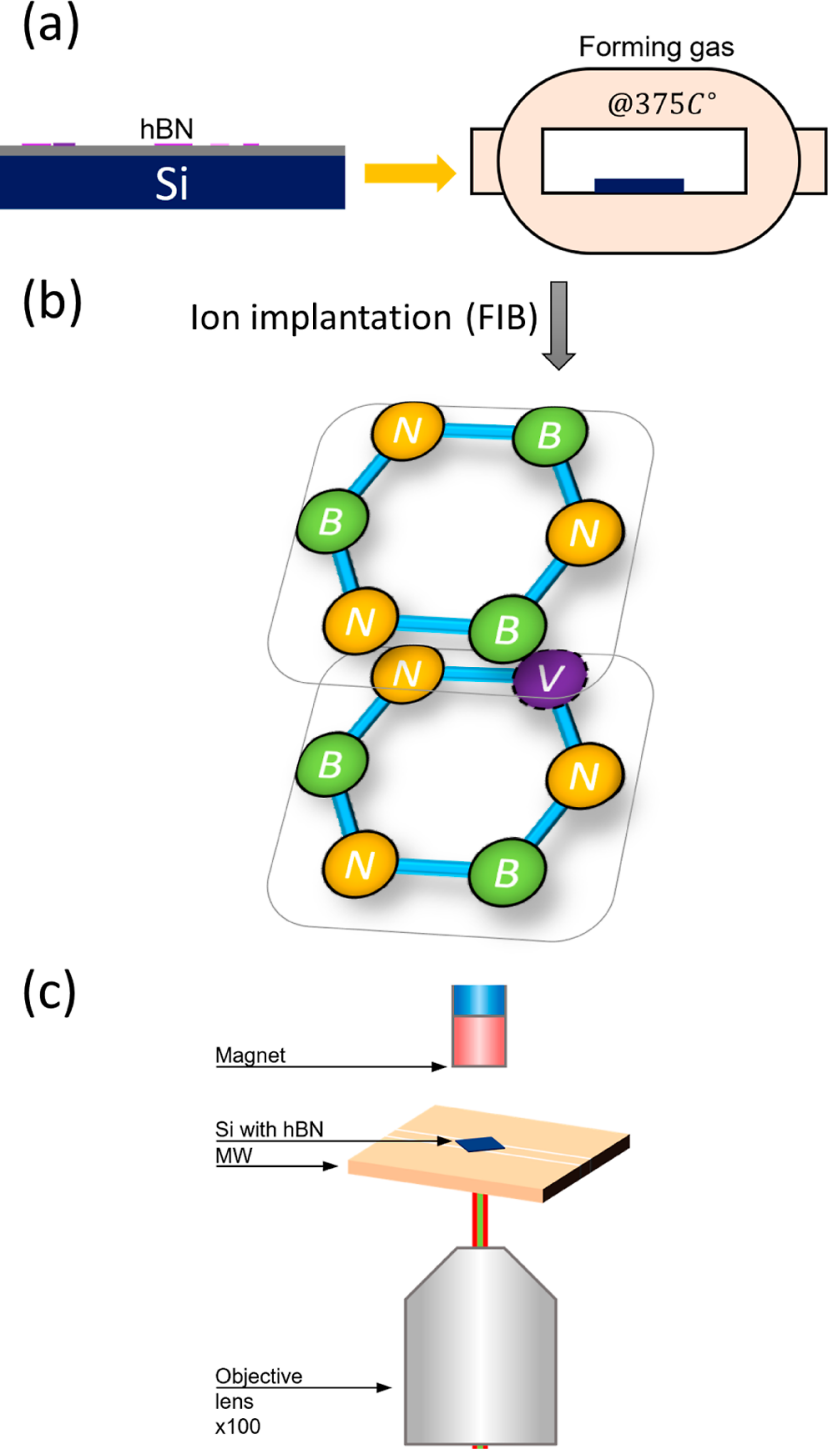
Schematic of the system, fabrication, and analysis. (a) hBN flakes exfoliated onto a Si substrate and treated in preparation for defect creation. (b) VB^–^ defects created controllably through local ion implantation using FIB. (c) Fluorescence and magnetic resonance analyses performed to characterize created defect properties.

In this work, we focused on the most commonly used approach for generating VB^–^ defects, namely, ion implantation, and specifically using a focused ion beam (FIB) to enable detailed systematic studies of the implantation parameters (with the capability of comparing different implantation parameters on the same flake). We studied the generation of VB^–^s using FIB in bulk exfoliated hBN, considering also the important effects of sample preparation and flake thickness on the yield of VB^–^s. We focused on sample thicknesses in the range of 50–200 nm, which is of interest due to the significant signal obtained above 50 nm, and the advantages of the vdW nature of the material below 200 nm (above which bulk-like behavior begins to emerge). We describe in detail the preparation method applied to the flakes, the methods applied for VB creation, and the characterization of the flakes by fluorescence spectroscopy and spin resonance measurements (Figure 1). We present the successful creation of VB^–^s using 12 keV ion implantation through FIB, with both nitrogen and oxygen ion beams. The defects were identified by their spectral signature with a peak emission at ∼800 nm and their spin resonance at ∼3.47 GHz at zero field.^17,18^ We observe increasing fluorescence with increasing ion fluence and identify a thickness dependence, such that thicker flakes show higher fluorescence intensities for the same fluence. Stopping range of ions in matter (SRIM) simulations were performed to better understand the defect creation process. Our results indicate that in practice, vacancies are created in the sample further than the ion stopping range, either due to ion channeling (which is not taken into account during the simulations) or more likely due to secondary ion processes. This extends the interaction depth of the ion beam with the samples, resulting in a thickness-dependent fluorescence of the flakes, as observed. Importantly, our systematic study extends previous results and identifies crucial aspects of sample preparation that strongly impact the robustness of defect creation as well as provides insights into the actual process of ion-induced defect creation through thickness-dependence results.

## 2 Sample Preparation

One of the most common and straightforward techniques for preparing hBN flakes is exfoliation from bulk crystal (using blue tape, Nitto), similar to the processes originally developed for graphene and molybdenum disulfide (MoS_2_).^1^ We note that for the purposes of this work, careful exfoliation in a clean environment is required, as otherwise carbon contaminants might be introduced into the flakes,^19^ which could then impede the creation of VB^–^s, introduce other defects to the material, and lead to decreased signal to noise ratio (SNR) for VB^–^ spin readout.

Once exfoliated, flakes were transferred to a silicon (Si) substrate with a thin layer (285 nm) of silicon dioxide (SiO_2_) and gold markers. To clean the flakes from contaminants that were introduced during exfoliation, the samples were heated in forming gas (95% Ar and 5% H) for 4 h at 375 °C. We performed this cleaning process on flakes marked N1, N2, and O1 and O2 (see Table 1 below). Additionally, another sample (marked flake N3) was cleaned using an open-air hot plate, for 15 min at 400 °C, with similar results. The details of the characterization for this flake (and a couple of others used to confirm the findings reported here) are included in the Supporting Information.

**Table 1:** Parameters of hBN Flakes Presented in This Work: Name, Thickness, Fluence, and Preparation Process

flake name	flake thickness [nm]	ion fluence [ion/cm^2^]	cleaning method	color in figures
N1	100/150	6.25×1014	forming gas	blue/orange
N2	73	dose test	forming gas	yellow
N3	N.A.	dose test	hot plate	N.A.
O1	114	1.8×1013	forming gas	red
O2	83	dose test	forming gas	green

Comparing flakes that underwent this preparation process to similar flakes that have not demonstrated that such heat-based cleaning is crucial for the robust creation of VB^–^ defects, even compared to lower temperature heating (up to 250 °C). We conclude that successful defect creation relies on careful heat-based preparation of the samples, which constitutes a significant result of this work.

## 3 Defect Creation

### 3.1 Ion Implantation

Ion implantation,^14^ was the focus of this work and was implemented on flakes N1, N2, N3, O1, and O2, summarized in Table 1. This approach, which resulted in our most successful VB^–^ yield, was based on using a focused ion beam (FIB, Helios 5 Hydra Dualbeam plasma FIB/SEM instrument), utilizing Nitrogen (N) and Oxygen (O) ion elements at 12 keV.

The local implantation afforded by the FIB machine afforded the realization of dose tests on specific flakes, which were performed with both oxygen and nitrogen ion beams (see Table 1). In both cases, the parameters used, ion beam energy, beam size, dwell time, and beam current, were kept constant, while changing the number of exposure repetitions to achieve varying dosages. We found that the VB^–^ yield is not sensitive to changing these parameters, as long as the total beam fluence is kept unchanged.

The fluence was calculated in the following way
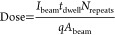
1where *I*_beam_ is the beam current, *t*_dwell_ is the dwell time, *N*_repeats_ is the number of repetitions, *q* is an electron’s charge, and *A*_beam_ is the beam area.

Flakes N1 and O1 underwent a uniform FIB exposure, with fluences of 6.25 × 10^14^ and 1.81 × 10^13^ cm^–2^ respectively, while the flakes N2, N3, and O2 underwent dose tests in an array of square regions of size 3 × 3 μm^2^.

We note that increasing the fluence further (see details in the Supporting Information), to the range of ∼10^16^ cm^–2^, creates visible damage on the flakes (observed optically as a change in the flake’s color), although VB^–^s were also created. Increasing the dose even further, up to 10^17^ to 10^18^ cm^–2^, tends to sputter away layers without creating any VB^–^s, essentially leading to unwanted damage to the flakes. We therefore focus our analysis on fluences up to ∼10^15^ cm^–2^, although we note that in principle there are additional factors that should be taken into account in this context, such as flake thickness and beam energy.

### 3.2 Additional Methods

The two main additional techniques for defect creation (beyond FIB ion implantation) are bulk (commercial) ion implantation and electron irradiation.

Commercial ion implantation is essentially equivalent to FIB, despite not enabling local control (e.g., dose tests on a single flake). We detail our bulk implantation tests in the Supporting Information. We note that in these tests and similar runs on the FIB, for which sample preparation did not include the process described above, VB creation was limited, and unwanted defects/contaminants appeared.

Electron irradiation is commonly used for defect creation and could be realized using standard electron fabrication/analysis tools: e-beam lithography and electron microscopy (although with limited energy). We carried out several experiments using these tools, detailed in the Supporting Information. The energy limitation was significant in our case, and therefore, VB creation was largely unsuccessful.

## 4 Sample Characterization

Following the above sample preparation protocols and defect creation schemes, we systematically characterize the flakes through their optical spectroscopic signatures to identify VB^–^ defects, their thickness to correlate with defect yield, and optically detected magnetic resonance (ODMR) to verify the spin properties of the defects.

### 4.1 Optical Spectroscopy

**Figure 2:**
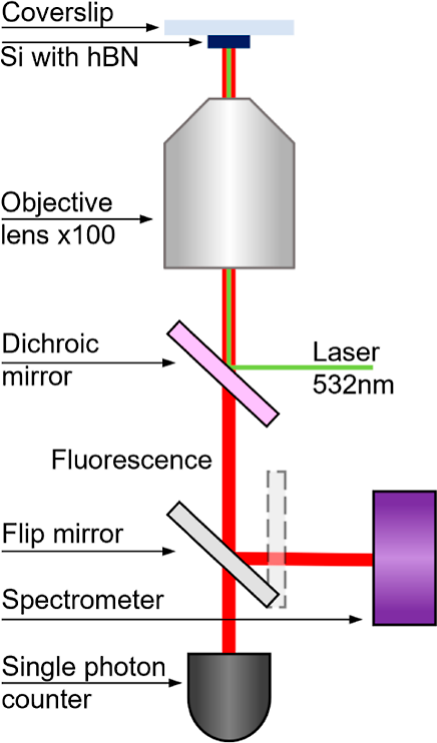
Confocal setup sketch for spectral measurements; 532 nm laser is used for excitation through an M Plan Apo 100×/0.95. PL is collected through a dichroic mirror either to a single photon counter (PerkinElmer SPCM, model AQRH-14) for confocal scanning, or to a spectrometer (Andor SR500i).

Given the well-known spectral signature of VB^–^ defects,^17^ optical spectroscopy is an efficient and convenient tool for characterizing defect creation. Emission spectra of the defects were measured using a homemade confocal setup (Figure 2) equipped with a spectrometer (Andor SR500i).

We note that photoluminescence (PL) spectral measurements provide high spatial resolution (submicron), yet lack the resolution to resolve depth details in our samples, which include thicknesses of <200 nm. As a result, we are insensitive to potential optical artifacts such as interference effects and varying focal planes. At the same time, we measure depth-integrated fluorescence and cannot identify nonuniform defect distributions throughout the thickness. For this reason, we measure the integrated fluorescence from flakes of varying thicknesses, while for each thickness we measure the dependence on implantation energy (which is related to implantation depth).

**Figure 3:**
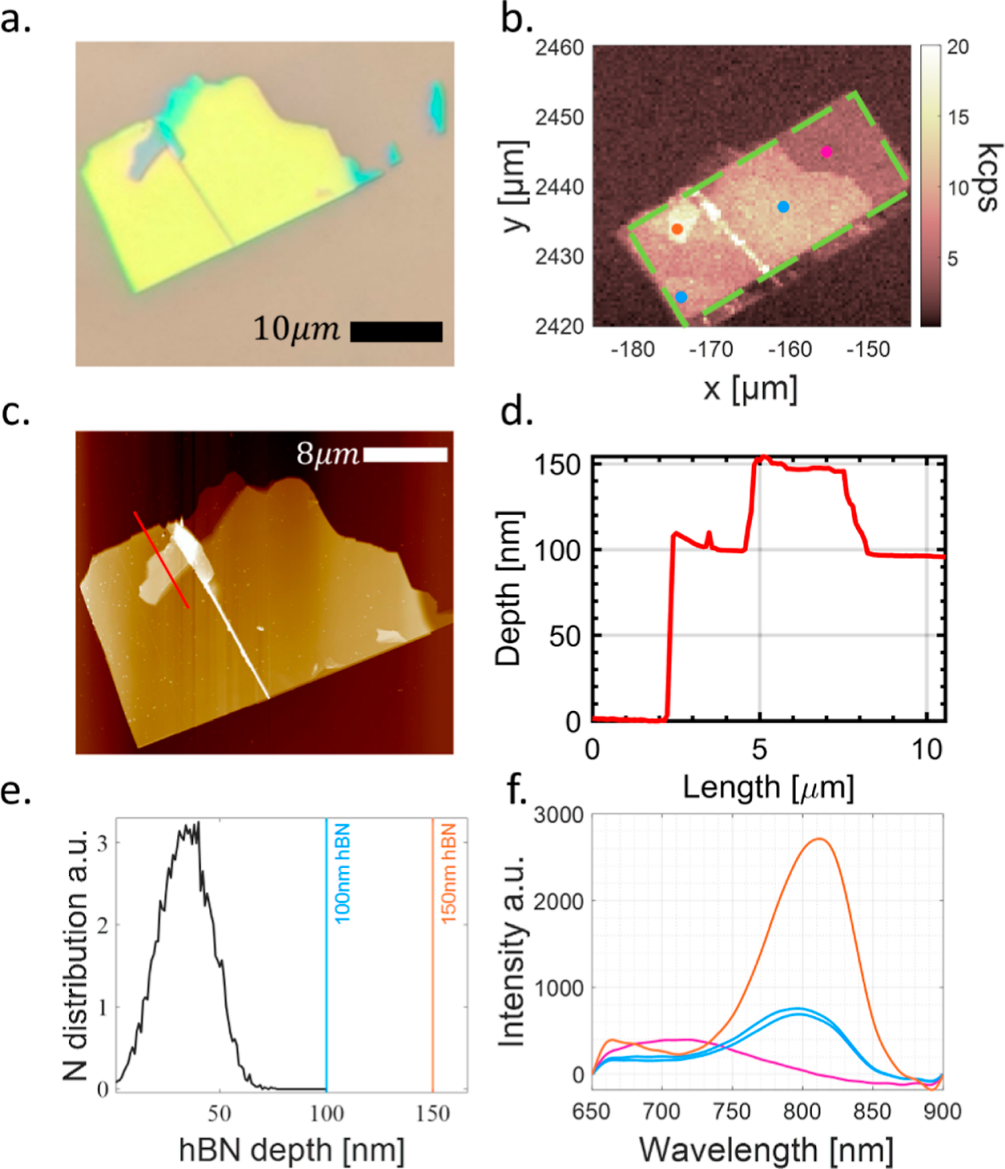
Analysis results for flake N1. (a) Optical microscopic image of the flake. (b) Confocal scan of the flake (borders marked with a green dashed line). The dots depict the areas for which spectral measurements were performed (see below). (c,d) AFM scan of the flake, depicting thickness variations in the relevant regions. The red line marks the 1D AFM thickness scan plotted in (d). (e) SRIM simulation showing the N ion depth distribution for energy of 12 keV, μ = 33.3 nm, and σ = 12.1 nm. (f) Comparison of the optical spectral signature of two thicknesses of the hBN—150 nm (orange), 100 nm (blue), and the silicone substrate for reference (pink).

In Figure 3 we analyze flake N1 (see Table 1). This flake was exposed to a uniform fluence of Nitrogen FIB implantation with the following parameters:

- beam size 0.01 μm^2^,
- dwell time 1 μs,
- current of 10 *pA*,
- exposure repetitions 1k.

These parameters translate to a dose of 6.25 × 10^14^ cm^–2^.

A microscope image of the flake and a confocal PL scan are shown in Figure 3a,b. A small fold in the upper left region of the flake indicates the potential for a region with a different thickness, which is confirmed by the atomic force microscopy (AFM) image (Figure 3c) and the scan along the red line, detailed in Figure 3d. The flake thus has relevant implanted regions with thicknesses of 100 and 150 nm, which we probe spectroscopically at the colored dots marked in Figure 3b. We performed simulations of the expected implantation depth (using SRIM—stopping range of ions in matter), depicted in Figure 3e, which indicates that this depth is significantly shallower than the flakes thicknesses, suggesting that VB creation should be similar for both. Spectral measurements confirm the creation of VB^–^s in flake N1 [Figure 3f]. The clear difference in the VB PL between spots of different thicknesses suggests that the naive analysis described above using SRIM is incorrect. Following this insight we studied the thickness dependence further (see below in Section 4.2), leading to one of the main results of this work.

The spectral measurements depicted in the figures were smoothed using triangle filters for visual purposes. Additionally, the spectral raw data were fitted to Gaussians in the relevant areas, to estimate the SNR from the VB^–^s, further details of the SNR calculation can be found in the Supporting Information. The strongest PL signal and best SNR were observed from the thicker area of the flake (150 nm, SNR ∼ 20), compared to a thinner area (100 nm, SNR ∼ 9) [Figure 3f]. While some area of the Si substrate was exposed as well, and exhibited some photoluminescence (PL), it was clearly unrelated to the relevant hBN defects, as can be observed from the spectral signature [Figure 3f].

**Figure 4:**
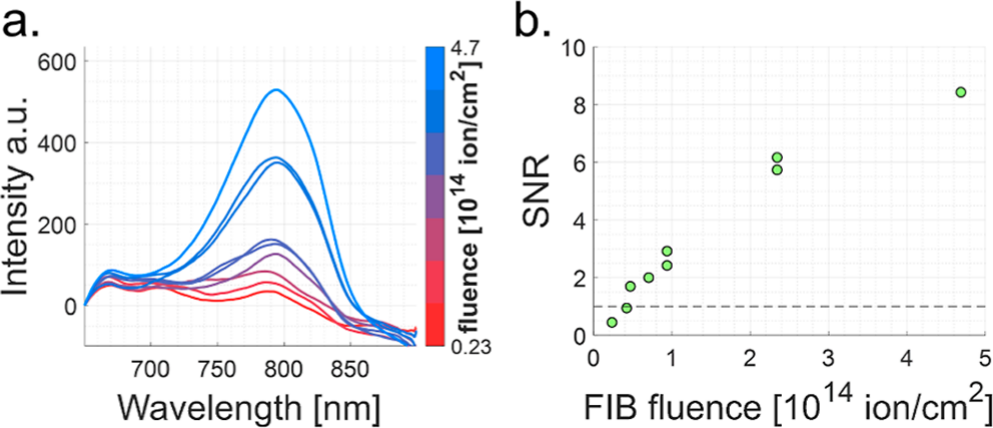
Measurements of PL spectra and analysis of oxygen implanted flake O2. (a) Measured PL spectra for different oxygen dosages realized on the flake through a FIB dose test (see color bar). (b) Extracted SNR of the VBs spectral signal, proportional to the yield of VB^–^ creation, as a function of dose. In these experiments (flake O2, thickness 83 nm, implantation energy 12 keV), the SNR (proportional to VB^–^ yield) grows monotonously (nearly linearly) with dose and finally starts to approach saturation. Note that the PL intensity is calibrated to zero at the shortest measured wavelength for comparative presentation, which does not affect our quantitative analysis.

Figure 4a depicts dose test experiments performed with Oxygen on flake O2 which is 83 nm thick, with the following parameters: energy 12 keV, beam size 0.04 μm^2^, dwell times of 1 μs and 10 μs, current of 30 *pA* and varying repetitions. The PL intensity increases monotonously with the dose with a nearly linear dependence (except for the onset of saturation appearing at the highest dose). Different dwell times do not have a significant impact on the PL intensity for the same dose.

### 4.2 Thickness Dependence

Following the results presented above for flake N1 at two different thicknesses, we characterized the optical spectra and defect creation yield for flakes of varying thicknesses and for both nitrogen and oxygen implantation.

**Figure 5:**
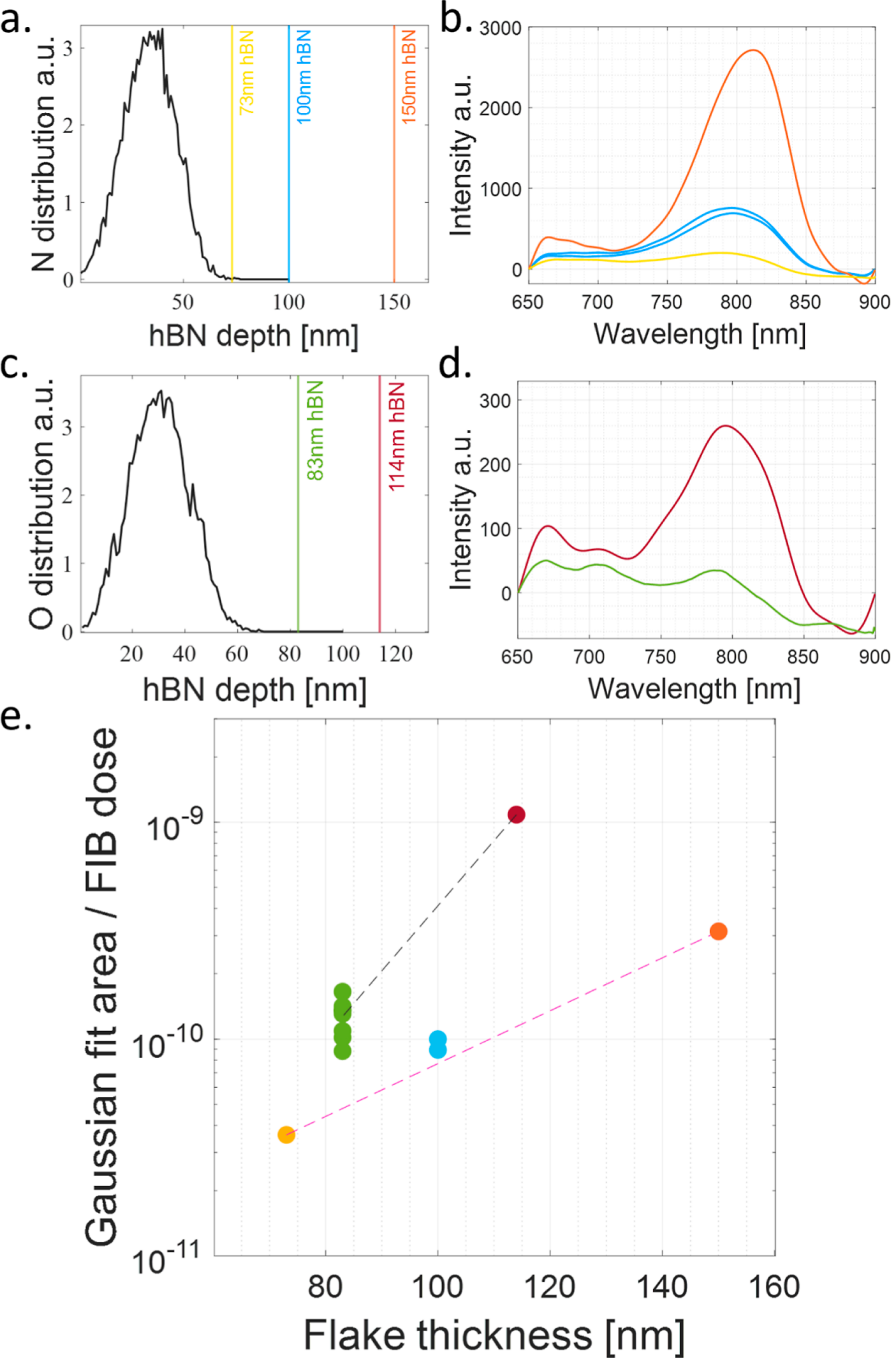
Thickness dependence of the VB^–^ creation yield under both nitrogen and oxygen FIB, measured through calibrated PL spectra compared to SRIM simulations. Comparative study of nitrogen implanted flakes N1 (100 and 150 nm, blue and orange) and N2 (73 nm, Yellow), and oxygen implanted flakes O1 (114 nm, red) and O2 (83 nm, green). (a) SRIM simulations for nitrogen implantation at 12 keV, with the relevant flake thicknesses marked. Note that the expected penetration depth is shallower than all flake thicknesses. (b) Comparison of PL spectral measurements for similar doses of nitrogen FIB ∼ 5 × 10^14^ cm^–2^. Thicker flakes present higher PL intensity, proportional to VB^–^ yield. (c) SRIM simulations for oxygen implantation at 12 keV, indicating shallower penetration compared to flake thicknesses (similar to nitrogen). (d) Comparison of PL spectral measurements of oxygen implanted flakes with a similar dose ∼ 2 × 10^13^ cm^–2^. Thicker flakes achieve higher PL intensity. (e) Calibrated dependence of VB^–^ creation yield vs flake thickness, for both nitrogen and oxygen FIB. We plot the integrated VB^–^ PL spectral signal normalized by the ion fluence (proportional to VB^–^ yield) vs flake thickness. Consistently thicker flakes achieve higher yields for both ion species nitrogen (dashed pink) and oxygen (dashed Black). Note that the PL intensity is calibrated to zero at the shortest measured wavelength for comparative presentation, which does not affect our quantitative analysis.

Figure 5 presents the results of our thickness dependence studies. We plot SRIM simulations of the ion penetration depth and the measured optical spectra for the different flakes for nitrogen implantation [subfigures (a) and (b)] and similarly for oxygen implantation [subfigures (c) and (d)].

For the nitrogen FIB, we see a clear difference in PL intensity [Figure 5b] and in calculated SNR, as a function of flake thickness [Figure 5e]. Similarly, PL spectral measurements on oxygen FIB implanted samples [Figure 5d,e] indicate that the thicker flake O1 (114 nm) has a VB^–^ creation yield (proportional to the measured SNR) approximately 8 times larger than the thinner flake O2 (83 nm), under similar FIB fluences.

It is clear from Figure 5a that the expected penetration depth for nitrogen at 12 keV is approximately 70 nm. Therefore it is not a-priori clear why for a given ion beam fluence, using a set energy of 12 keV, flakes thicker than 70 nm would show stronger PL and better SNR as a function of thickness.

Thus we conjecture that this result suggests that mainly ion channeling or secondary ions create the VB^–^ defects rather than first collision ions. Since angled implantation produces similar results, we expect that secondary ion collisions are the dominant defect creation mechanism. This constitutes a significant result of our work. We note that channeling effects have been observed for NV^–^s in diamonds,^20^ which also highlights the limitations of SRIM in properly predicting defect creation depths.

For oxygen implantation, for which we studied the thickness dependence (Figure 5) as well as performed a does test (Figure 4), we can quantify that in order to reach the same VB^–^ creation yield in the thin flake (O2, 83 nm) as the thick flake (O1, 114 nm), an increase of approximately an order of magnitude in FIB fluency is required.

These results indicate that SRIM simulations do not accurately predict the depth of VB^–^ creation in hBN, suggesting the important role of secondary ions in the creation of these defects. Practically, to achieve a specific VB^–^ yield in a given hBN flake, for a certain beam energy, the implantation dose should be calibrated with respect to the flake’s thickness since thicker flakes require a lower ion dose, compared to thinner ones.

### 4.3 Optically Detected Magnetic Resonance

To complement the measurements detailed thus far, we performed magnetic resonance measurements of the created VB^–^ defects. The magnetic resonance signature of these defects is further confirmation of their character. The VB^–^, like NV^–^, is a spin one system with a triplet ground state. Its resulting zero field splitting is ∼3.47 GHz.^17,18^ As such, and given its optical response, an optically detected magnetic resonance (ODMR) measurement can be performed to observe and characterize the VB^–^ spin properties.^21^

The pulsed ODMR sequence consists of an optical pumping pulse to initialize the spins to their *m*_s_ = 0 state and a microwave (MW) pulse applied with varying frequencies, followed by a final optical pulse for spin readout. At the spin resonant MW frequency, spin transitions are induced between the *m*_s_ = 0 and *m*_s_ = ± 1 ground states, such that the resulting fluorescence is suppressed.

**Figure 6:**
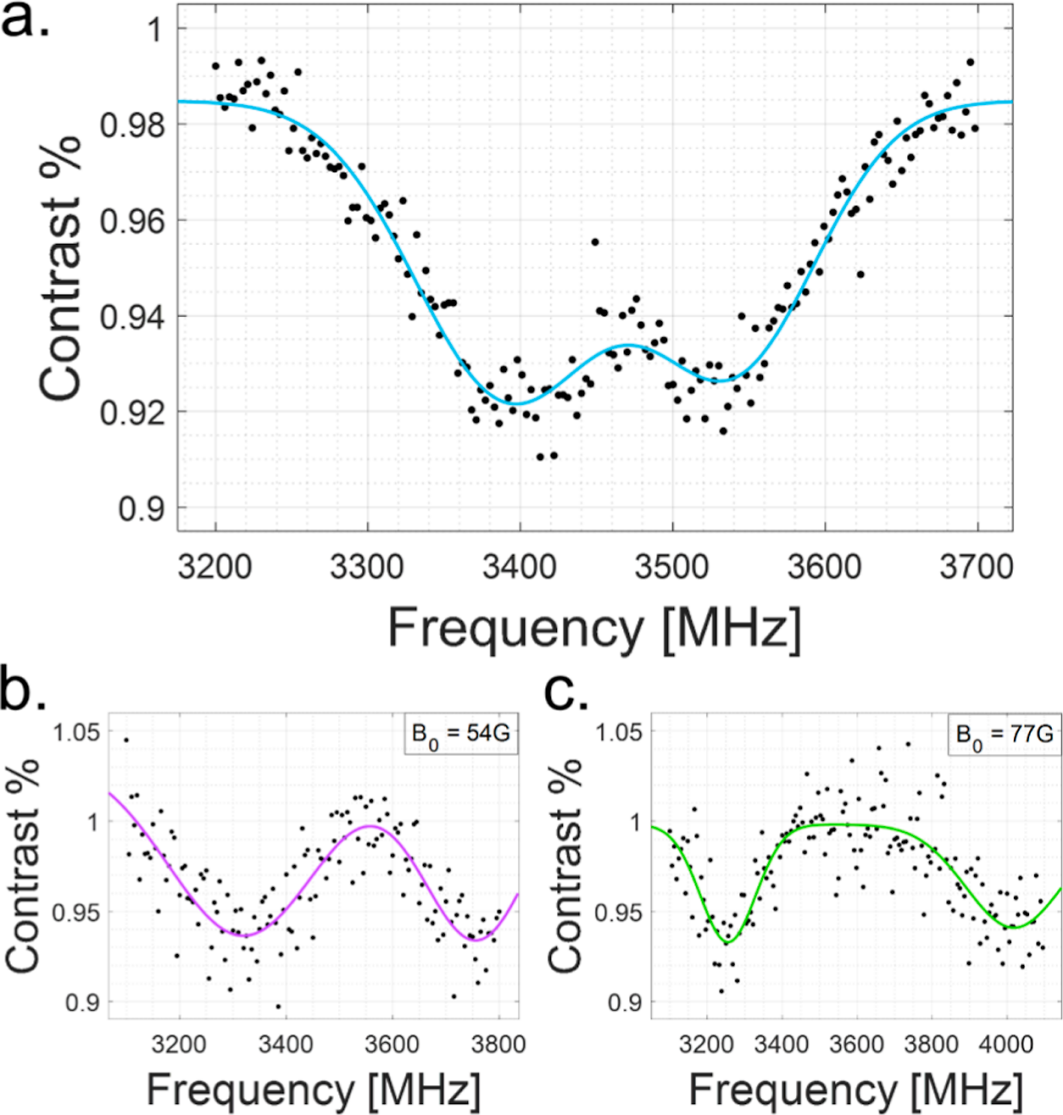
Optically detected magnetic resonance measurements on flake N1, demonstrating the expected spin resonance response of the created VB^–^ defects, as a function of externally applied magnetic field (depicting linear Zeeman shifts consistent with the expected  dependence). (a) Zero Bias field. (b) Bias field of 54 G and (c) 77 G.

To perform the ODMR we used an Ω-shaped coplanar antenna, ensuring that the flake is as close as possible to the conducting loop to achieve efficient MW driving. Zero field ODMR measurements on the nitrogen FIB implanted flake N1 are presented in Figure 6a. Additional ODMR measurements with an externally applied magnetic field at 54 and 77 G are presented in Figure 6b,c, demonstrating the linear Zeeman splitting between the VB^–^ ground state spin sublevels *m*_s_ = ±1.

Similar results were achieved on oxygen FIB flakes (Supporting Information), exhibiting ODMR contrast variations with PL intensity for the different flakes, related to different VB^–^ concentrations.

## 5 Summary and Conclusions

We have presented a study of the application of ion implantation, namely, nitrogen and oxygen FIB (focused ion beam) implantation, for the robust creation of VB^–^ defects in hBN flakes. Despite the growing interest in solid-state, optically active spin defects and specifically the promising VB^–^ defects in 2D layers of the van der Waals (vdW) material hBN, such a systematic study was missing.

Our results show that both oxygen and nitrogen ions at 12 keV can efficiently create VB^–^s in hBN flakes of various thicknesses. Our two main findings include the following: (i) we find that hBN flake thickness plays a key role in the success of the defect creation process and in the achievable VB^–^ yield. Specifically, we find that secondary ion collisions are dominant in creating VB^–^ defects with defect penetration reaching well beyond that expected through SRIM simulations. Moreover, for a given beam energy, a higher dose is required to create the desired defect yield for thinner flakes. (ii) We find that the cleaning process used on the flakes prior to implantation is crucial. Specifically, the inclusion of a forming gas heating step or hot plate cleaning at approximately 400C proved important for robust creation of VB^–^ in hBN.

As a useful parameter set for VB creation, addressing nitrogen implantation with 12 keV and hBN flakes approximately 100 nm thick, a dose of 5 × 10^14^ [cm^–2^] achieves a relatively high SNR defect response, with an estimated lateral concentration of ∼10^14^ [cm^–2^] VBs.

We also note that in certain cases flakes could be created by well-controlled, clean methods not related to exfoliation.^22,23^ Nevertheless, exfoliation from bulk crystals is a widely used technique, with the advantage of providing access to high-quality material. As such, the techniques presented here and, specifically, the cleaning processes should be carefully considered prior to implantation.

## Supporting Information Available

Includes additional details on sample preparation, data analysis, electron irradiation, ion implantation, and further measurement data on FIB fluence and thickness dependence through PL spectra and magnetic resonance response (PDF)
