# Supplementary material for: Creation of Boron Vacancies in Hexagonal Boron Nitride Exfoliated from Bulk Crystals for Quantum Sensing
Source: ACS Appl Nano Mater. 2023 Nov 28;6(23):21671–8. doi: 10.1021/acsanm.3c03395 (PMC11145586; doi:10.1021/acsanm.3c03395)
Supplement: Supplementary file 1 — an3c03395_si_001.pdf [file an3c03395_si_001.pdf]

Supporting Information: Creation of Boron Vacancies in Hexagonal Boron Nitride Exfoliated from Bulk Crystal for Quantum Sensing

Ty Zabelotsky,[†,‡] Sourabh Singh,[¶] Galya Haim,[‡,§] Rotem Malkinson,[†,‡] Shima Kadkhodazadeh,[‖] Ilya P. Radko,[⊥] Igor Aharonovich,[#,@] Hadar Steinberg,[†,¶] Kirstine Berg-Sørensen,[△] Alexander Huck,[∇] Takashi Taniguchi,[††] Kenji Watanabe,[‡‡] and Nir Bar-Gill[*,†,‡,¶]

†*The Center for Nanoscience and Nanotechnology, The Hebrew University of Jerusalem, Jerusalem 91904, Israel*

‡*The Institute of Applied Physics, The Hebrew University of Jerusalem, Jerusalem 91904, Israel*

¶*The Racah Institute of Physics, The Hebrew University of Jerusalem, Jerusalem 91904, Israel*

§*School of Physics, The University of Melbourne, Parkville, Victoria 3010, Australia*

‖*DTU Nanolab, Technical University of Denmark, Fysikvej, Kongens Lyngby 2800, Denmark*

⊥*Department of Physics, Technical University of Denmark, Kongens Lyngby 2800, Denmark*

#*School of Mathematical and Physical Sciences, University of Technology Sydney, Ultimo, New South Wales 2007, Australia*

@*ARC Centre of Excellence for Transformative Meta-Optical Systems (TMOS), Faculty of Science, University of Technology Sydney, Australia*

△*Department of Health Technology, Technical University of Denmark, Kongens Lyngby 2800 Denmark*

∇*Center for Macroscopic Quantum States (bigQ), Department of Physics, Technical University of Denmark, 2800 Kongens Lyngby, Denmark*

††*International Center for Materials Nanoarchitectonics, National Institute for Materials Science, 1-1 Namiki, Tsukuba 305-0044, Japan*

‡‡*Research Center for Functional Materials, National Institute for Materials Science, 1-1 Namiki, Tsukuba 305-0044, Japan*

E-mail: nir.bar-gill@mail.huji.ac.il

1 Sample Preparation - Hot plate

As mentioned in the main text, some samples were cleaned using a hot plate set to $400°C$ for 15 minutes. This process, while less controlled than the forming gas heating, is widely available and straightforward to implement, and thus of interest.

In Figure S1 we present PL spectral measurements performed on flake N3, which was prepared using the hot plate method and ex-

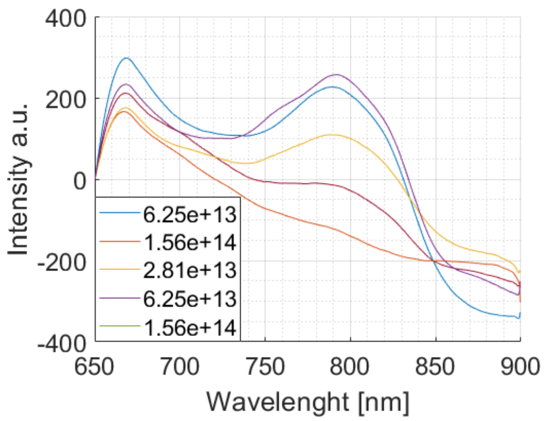

Figure S1: Flake N3 dose test, varying fluences in the range of $10^{13} \sim 10^{14} cm^{-2}$

posed to a nitrogen FIB dose test. These results indicate efficient creation of VB^- defects, comparable to the yield obtained using forming gas sample preparation. The hot plate cleaning process did not eliminate all contaminants from the flakes, thus some fluorescence is visible for wavelengths below 700 nm, similarly to flakes that went though annealing in forming gas.

We propose that the contaminants might have been introduced during a SEM imaging session, though this statement was not thoroughly tested and cannot be fully confirmed.

2 Defect creation - Additional Techniques

2.1 Bulk Ion Implantation

Bulk ion implantation was attempted using standard, commercial (semiconductor industry) implanter instrumentation (Innovion Corp.) to reproduce previously reported results.[S1] In our case these trials were not successful, resulting in measured fluorescence but not with characteristics matching those of VB^-s (Fig. S2). We attribute the measured fluorescence to other defects. Nevertheless, we note that for this process no cleaning was performed on the flakes post transfer to the Si substrate (before implantation). Since unclean flakes exhibited limited defect creation also using FIB, we deduce that sample cleaning before implantation plays an important role.

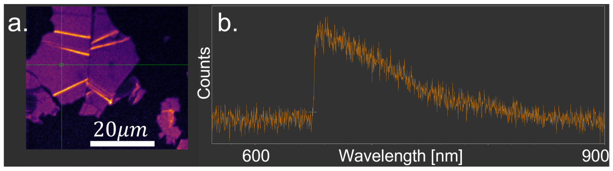

Figure S2: Two flakes that underwent uniform fluence of ion beam $10^{14} cm^{-2}$ at 40 keV. (a) confocal scan. (b) PL spectra measured in these flakes, depicting no VB^- signature.

2.2 Electron Irradiation

Furthermore, we studied electron irradiation instead of ion implantation, performed using two electron sources: e-beam lithography (100keV, Elionix ELS-G100) and TEM (80keV and 300keV, Themis-Z). The hBN samples used for these electron irradiation studies went through the same sample preparation as detailed for the FIB. For TEM irradiation, the flakes were transferred to TEM Gold grids (200 Mesh) with carbon film (R 1.2/1.3) using the following procedure: first, hBN flakes were transferred to a Si substrate spin coated with Poly(vinyl alcohol) (PVA), then the TEM grid's carbon side was placed on the Si, and finally a drop of water was added to dissolve the PVA, allowing the hBN flakes to be transferred to the grid.

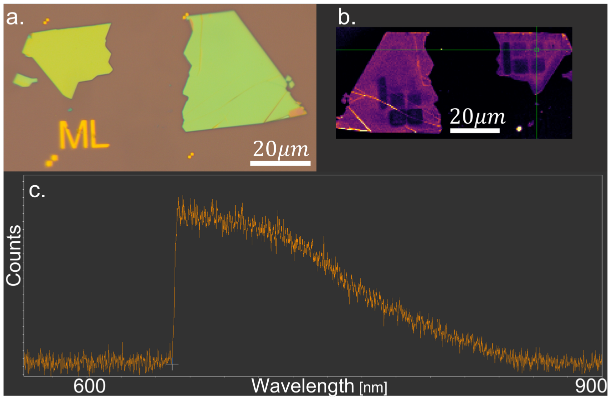

Figure S3: Two flakes that underwent e-beam dose test ranging from $10^{17} cm^{-2}$ to $10^{19} cm^{-2}$ at 100 keV. (a) Microscope image of the flakes. (b) Confocal scan of the fluorescence from the flakes after the irradiation. (c) PL Spectra of exposed areas on the flakes, depicting no VB^- signature.

Electron irradiation under the above conditions did not result in successful creation of VB^-s, as can be seen in Fig. S3 for e-beam and in Fig. S4 for TEM. We attribute this to insufficient energy of the electron beam. Theoretically, the maximum energy that 80 keV and 100 keV electrons can transfer to B atoms are approximately 17.5 eV and 22.2 eV, respectively.[S2,S3] Given the displacement threshold of 19.36 eV reported for B in pristine hBN,[S4] it is unlikely to create VB^-s in hBN flakes using 80 keV electrons. Although the energy transfer from 100 keV electrons should be sufficient for VB^-s creation, the probability of such a process is relatively low and likely requires very high electron beam doses.[S4]

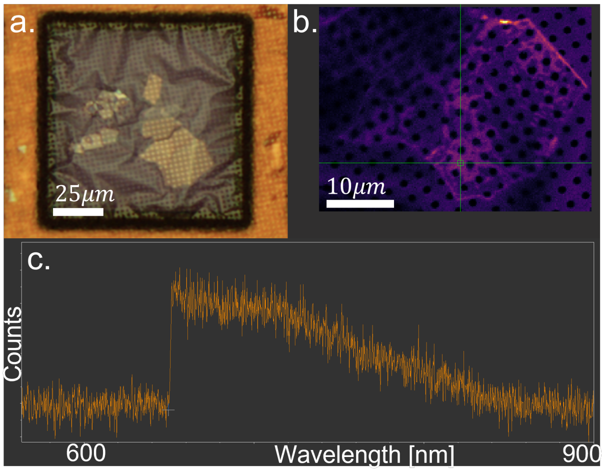

Figure S4: Two flakes that underwent TEM dose test ranging from $10^{19} cm^{-2}$ to $10^{22} cm^{-2}$ at 300 keV. (a) Microscope image of the flakes. (b) Confocal scan of the fluorescence from the flakes after the irradiation. (c) PL spectra of exposed areas on the flakes, depicting no VB^- signature.

3 Data smoothing and SNR

The PL spectral data was smoothed using standard low-pass filtering, for enhanced presentation clarity and for quantitative SNR and VB yield analysis. The raw data was smoothed out using a triangular low-pass filter as follows:

$$S(\lambda) = \frac{\sum_{m=-\lfloor \frac{w}{2} \rfloor}^{\lfloor \frac{w}{2} \rfloor} (\lceil \frac{w}{2} \rceil - |m|) Y(\lambda + m \cdot \Delta\lambda)}{w + 2 \cdot \sum_j (w - j)}, \tag{1}$$

where $S(\lambda)$ is the output, smoothed signal, $\Delta\lambda = 0.2\,\text{nm}$ is the wavelength increment, $Y(\lambda)$ the raw data and $w = 100$ is the size of the smoothing filter.

The SNR of the VB^- signal, proportional to the VB^- yield, is estimated from the data, by fitting a Gaussian to the raw PL spectral data (see Fig. S5) using the following formula:

$$fit = A \cdot \exp{(-\frac{(\lambda - \mu)^2}{2 \cdot \sigma^2})} + m \cdot x + C. \tag{2}$$

We excluded data points below 650 nm, since the relevant data is above this threshold, and technically a high-pass filter at that wavelength was used during acquisition.

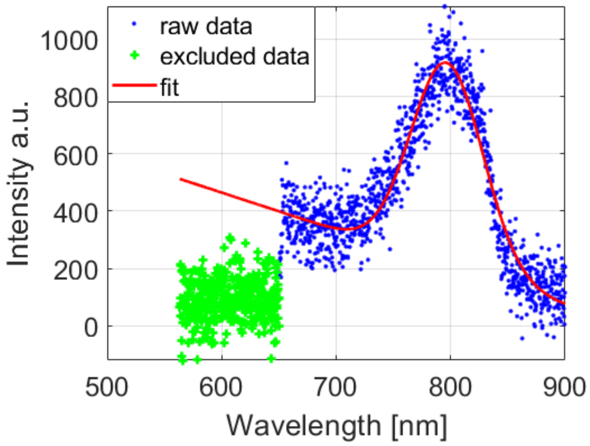

Figure S5: Spectral measurement from flake N1 with a fit to the Gaussian model to extract the VB^- SNR (proprtional to defect yield): $A \cdot \exp{(-\frac{(\lambda-\mu)^2}{2 \cdot \sigma^2})} + m \cdot x + C$

Finally for the SNR calculation, we extract the ratio between the area under the Gaussian and root mean square error (σ_{RMSE}) of the fit:

$$SNR = \frac{A \cdot \int \exp{(-\frac{(\lambda-\mu)^2}{2 \cdot \sigma^2})} d\lambda}{\sigma^2_{RMSE}} \tag{3}$$

4 Additional Data

4.1 FIB Fluence and Thickness Dependence

We perfromed additional experiments to further verify our results, including dose tests and thickness dependence studies, analyzing the PL spectral signatures from the various implanted regions as described in the main text.

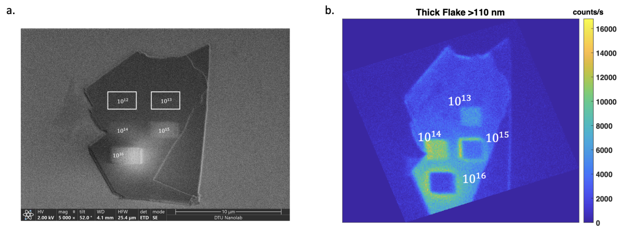

Figure S6: Additional dose test studies using nitrogen FIB, presented on 110 nm thick flake. (a) SEM image. (b) Confocal scan depicting the PL from the different implant regions.

In Fig. S6 we present SEM and confocal images of a flake implanted with nitrogen FIB dose test. Such studies were performed on several additional flakes of varying thicknesses. As the dose of $\sim 10^{14}[cm^{-2}]$ proved most efficient in this range, we analyzed the VB^{-} yield for this dose as a function of thickness, as presented in Fig. S7. These results are consistent and complement the data and analysis presented in the main text.

4.2 Optically Detected Magnetic Resonance (ODMR)

In addition to the magnetic resonance measurements presented in the main text, we have also characterized the created VB^{-} defects in the oxygen FIB samples. A representative ODMR measurement is shown in Fig. S8, demonstrating the signature resonance feature of VB^{-} defects.

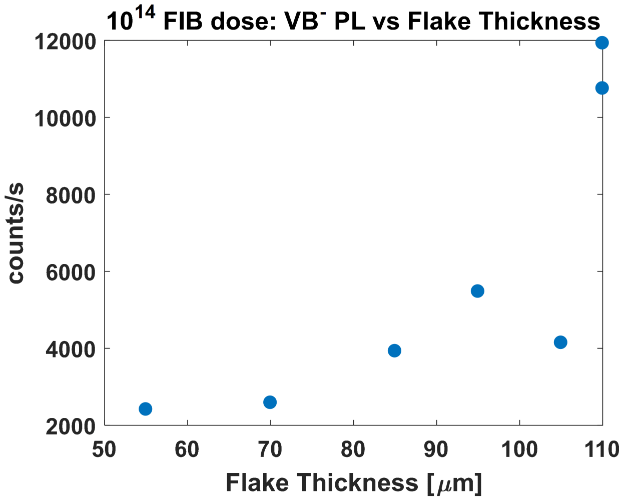

Figure S7: Additional thickness dependence analysis for nitrogen FIB implantation at 12 keV and a dose of $10^{14}[cm^{-2}]$. These results are consistent and complement the analysis presented in the main text.

References

(S1) Guo, N.-J.; Liu, W.; Li, Z.-P.; Yang, Y.-Z.; Yu, S.; Meng, Y.; Wang, Z.-A.; Zeng, X.-D.; Yan, F.-F.; Li, Q.; Wang, J.-F.; Xu, J.-S.; Wang, Y.-T.; Tang, J.-S.; Li, C.-F.; Guo, G.-C. Generation of Spin Defects by Ion Implantation in Hexagonal Boron Nitride. *ACS Omega* **2022**, *7*, 1733–1739.

(S2) Egerton, R. F. *Electron Energy-Loss Spectroscopy in the Electron Microscope; 3rd ed*; Springer New York, NY, 2011.

(S3) Egerton, R.; Li, P.; Malac, M. Radiation damage in the TEM and SEM. *Micron* **2004**, *35*, 399–409.

(S4) Kotakoski, J.; Jin, C. H.; Lehtinen, O.; Suenaga, K.; Krasheninnikov, A. V. Electron knock-on damage in hexagonal boron nitride monolayers. *Phys. Rev. B* **2010**, *82*, 113404.

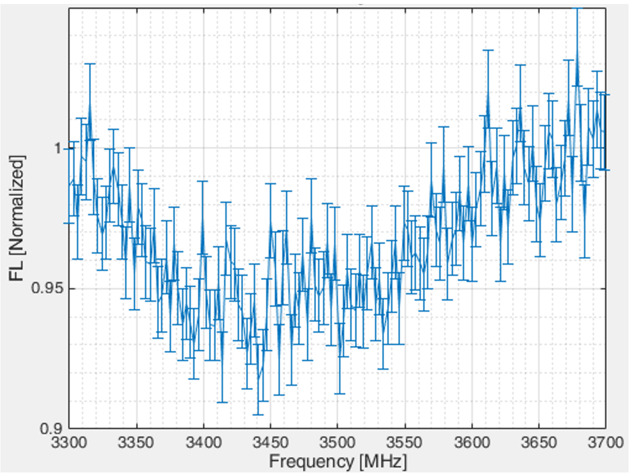

Figure S8: ODMR measurement performed on flake O1, implanted with oxygen FIB, without a bias magnetic field. The magnetic resonance signature of VB^- is clearly visible.
